# Home healthcare services in Taiwan: a nationwide study among the older population

**DOI:** 10.1186/1472-6963-10-274

**Published:** 2010-09-21

**Authors:** Hsiao-Ting Chang, Hsiu-Yun Lai, I-Hsuan Hwang, Mei-Man Ho, Shinn-Jang Hwang

**Affiliations:** 1 Department of Family Medicine, Taipei Veterans General Hospital, No. 201, Sec. 2, Shipai Rd., Beitou Dist., Taipei City, 11217, Taiwan; 2 Center for Geriatrics and Gerontology, Taipei Veterans General Hospital, No. 201, Sec. 2, Shipai Rd., Beitou Dist., Taipei City, 11217, Taiwan; 3 National Yang-Ming University, School of Medicine, No. 155, Sec. 2, Linong St., Taipei City, 11221, Taiwan; 4 Shih Chien University, College of Management, No.70, Dazhi St., Taipei City, 10462, Taiwan; 5 Taipei Veterans General Hospital Home Care Institute, No. 201, Sec. 2, Shipai Rd., Taipei City, 11217, Taiwan

## Abstract

**Background:**

Home healthcare services are important in aging societies worldwide. The present nationwide study of health insurance data examined the utilization and delivery patterns, including diagnostic indications, for home healthcare services used by seniors in Taiwan.

**Methods:**

Patients ≥65 years of age who received home healthcare services during 2004 under the Taiwanese National Health Insurance Program were identified and reimbursement claims were analyzed. Age, gender, disease diagnoses, distribution of facilities providing home healthcare services, and patterns of professional visits, including physician and skilled nursing visits, were also explored.

**Results:**

Among 2,104,978 beneficiaries ≥65 years of age, 19,483 (0.9%) patients received 127,753 home healthcare visits during 2004 with a mean number of 6.0 ± 4.8 visits per person. The highest prevalence of home healthcare services was in the 75-84 year age group in both sexes. Females received more home healthcare services than males in all age groups. Cerebrovascular disease was the most frequent diagnosis in these patients (50.7%). More than half of home healthcare visits and around half of the professional home visits were provided by community home nursing care institutions. The majority of the home skilled nursing services were tube replacements, including nasogastric tubes, Foley catheter, tracheostomy, nephrostomy or cystostomy tubes (95%).

**Conclusions:**

Nine out of 1,000 older patients in Taiwan received home healthcare services during 2004, which was much lower than the rate of disabled older people in Taiwan. Females used home healthcare services more frequently than males and the majority of skilled nursing services were tube replacements. The rate of tube replacement of home healthcare patients in Taiwan deserves to be paid more attention.

## Background

Population ageing with an increasingly disabled population has become a major concern in developed and developing countries [1,2], and the demand for long-term care services is rising [1-3]. Taiwan became a World Health Organization (WHO) defined ageing society in 1993, and the percentage of population aged ≥65 years (an aged population) is predicted to double by 2017 [4]. The impact of rapid population ageing is complex and raises many healthcare issues including the long-term care of disabled people in countries including Taiwan [5].

Home healthcare is one part of a continuum of health care in many countries. In Taiwan, home healthcare services are reimbursed by the National Health Insurance (NHI) program [6]. According to the Home Nursing Care Payment Regulations in Taiwan, home healthcare service reimbursements are limited to medical or nursing institutions that meet the requirements of the Department of Health (DOH). Furthermore, the regulated parameters include the frequency of professional visits including physician visits (one visit every two months) and nursing visits (one visit every two weeks) [7]. In Taiwan, becoming a practice home healthcare nurse requires registered nurses to complete basic training courses including curricula on long-term care, infection control, disabled care, nutritional care, physical examination, terminal care, swallowing and speech care, and maintenance of activities of daily living. Clinical practical internships in home healthcare and in long-term care facilities are also needed [8]. A patient qualified to apply for home healthcare services needs to fulfill the following three criteria: 1) limited ability of self-care (over 50% of the time while awake, the patient is chair-bound or bed-bound), 2) definite medical or nursing care needs, and 3) chronic conditions requiring long-term nursing care, or continual nursing care needs following hospital discharge.

The home healthcare services items reimbursed by NHI in Taiwan include general nursing services, special skilled nursing services, laboratory tests, nurse visits and physician visits [7]. The general nursing care services include physical evaluations, nursing instructions, drug injections and fecal extraction. Special skilled nursing services include the changing of urinary catheters or nasogastric tubes, or tube tracheostomy, nephrostomy or cystostomy catheters; changing the dressing of stage III and IV pressure sores; intravenous fluid injection and ostomy nursing [7]. The NHI reimburses home nursing visits with fixed rates according to resource utilization groups (RUGs) graded 1-4 as follows: RUG-1 are patients who need general nursing care services only; RUG-2 patients need a single additional kind of special skilled nursing care service besides general nursing care; RUG-3 patients need two additional kinds of special skilled nursing care services; and RUG-4 patients need an additional three kinds of special nursing care service [7]. The NHI also reimburses physician visits in a fixed payment, not according to the RUG classification of patients as for nursing visits [7].

More information concerning home healthcare service utilization is needed to meet the needs of a rapidly aging population in Taiwan. Presently, a nationwide study of health insurance data was undertaken to examine utilization and delivery patterns, including diagnostic indications, for home healthcare services used by seniors in Taiwan.

## Methods

### Data sources

The NHI program, which was initiated in Taiwan in 1995, covered 22,143,270 beneficiaries of the population of 22,689,122 (97.6% coverage) inhabitants at the end of 2004 [9]. Since 1999, the Bureau of National Health Insurance has released the claims data to the National Health Research Institutes (NHRI) for research use under the NHI Research Database project. The structure of the claims datasets is described in detail on the NHRI website and in our previous publications [10-12].

All outpatients service visiting claims datasets for beneficiaries ≥65 years of age in 2004 (CD2004.DAT) were obtained. These datasets contained the home care visits, ambulatory care clinics and emergency department visiting files. Visiting data included dates, medical care facilities, genders, dates of birth, and the three major diagnoses for visits coded in International Classification of Disease, Ninth Revision, Clinical Modification (ICD-9-CM) format. The corresponding order files (OO2004.DAT) were also obtained; these contained details of management, including medical services, medical procedures, special medical materials used, laboratory tests, and prescribed medications presented by a unique coding number in each visit. The complete database of coding numbers for corresponding orders was obtained from the NHI website [13]. Another file obtained, HOSB2004.DAT, contained basic data about the healthcare facilities providing the accreditation levels: academic medical center, metropolitan regional hospital, local community hospital, primary care clinics, and home care institutions. All data identifying beneficiaries, physicians and institutions were encrypted to ensure privacy.

### Analyses

The database software, Microsoft SQL Server 2008 (Microsoft, Redmond, WA, USA), was used for data linkage, processing and computation. The results were presented by descriptive statistics.

## Results

At the end of 2004, a total of 2,104,978 beneficiaries aged ≥65 years of age were insured by the NHI program in Taiwan. Of these, 1,044,182 (49.6%) were female and 1,060,796 (50.4%) were male. From these beneficiaries, 19,483 (0.9%) patients (8,439 males and 11,044 females) were identified who had received a total of 127,753 home healthcare visits during 2004. The mean age of the homecare recipients was 79.1 ± 8.0 years for males and 80.1 ± 7.6 years for females. When stratified by age, the highest home healthcare service utilization was in those aged ≥85 years (3.4%) and the lowest was in those aged 65-74 years (0.4%). Females were more likely to receive home healthcare visits than males (1.1% vs. 0.8%, *P *< 0.0001), especially those aged 75-84 years (1.6% vs. 1.1%, *P *< 0.0001) and ≥85 years of age (3.9% vs. 2.7%, *P *< 0.0001). The gender difference increased with age (Table 1).

**Table 1:** Age-sex prevalence of patients receiving NHI funded home healthcare services in 2004.

No. of home healthcare patients* (% of beneficiaries)				No. of home healthcare visits* (%)			Home healthcarevisits per patient* mean ± SD	
**Age**	**Male**	**Female**	**Total, n (%)**	**Male**	**Female**	**Total, n (%)**	**Male**	**Female**

65-74	2,544 (0.4)	2,756 (0.4)	5,300 (0.4)	16,880 (13.2)	19,682 (15.4)	36,562 (28.6)	6.6 ± 4.7	7.1 ± 4.9
75-84	4,100 (1.1)	5,133 (1.6)	9,233 (1.3)	24,536 (19.2)	35,548 (27.8)	60,084 (47.0)	6.0 ± 4.6	6.9 ± 5.0
≥85	1,795 (2.7)	3,155 (3.9)	4,950 (3.4)	10,397 (8.2)	20,710 (16.2)	31,107 (24.4)	5.8 ± 4.6	6.6 ± 4.9
Total	8,439 (0.8)	11,044 (1.1)	19,483 (0.9)	51,813 (40.6)	75,940 (59.4)	127,753 (100)	-	-

*P < 0.0001, between males and females in the columns.

**Figure 1:**
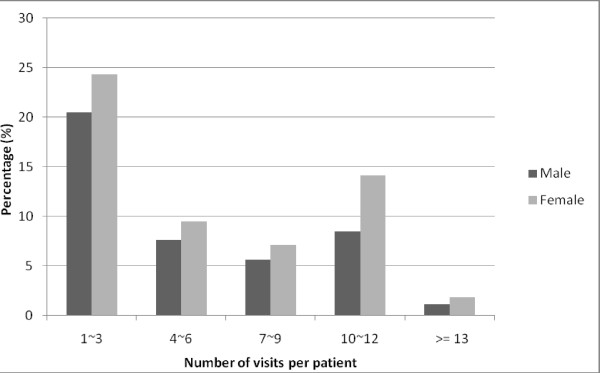
**Sex-specific frequency distribution for home healthcare visits per patient in 2004 (a total of 127,753 visits by 19,483 patients)**.

Considering the total home healthcare visits, individuals aged 75-84 years accounted for the greatest proportion of total visits (47.0%), while those ≥85 years of age represented the lowest proportion of visits (24.4%). On average, those aged 65-74, 75-84 and ≥85 years of age had 6.9 ± 4.8, 6.5 ± 4.8 and 6.3 ± 4.8 home healthcare visits, respectively (Table 1). The number of home healthcare visits per patient during 2004 was further arranged into five categories (1-3, 4-6, 7-9, 10-12 and ≥13 visits). Females were more likely to receive home healthcare visits than males in every category (Figure 1).

According to the ICD-9-CM coding systems, the five major diagnoses for patients receiving home healthcare visits were cerebrovascular diseases (n = 64,789, 50.7%), dementias (n = 14,432, 11.3%), diabetes mellitus (n = 7,523, 5.9%), other diseases of the lung (n = 3,755, 2.9%) and Parkinson's disease (n = 3,482, 2.7%).

Among all home healthcare visits to beneficiaries ≥65 years of age in 2004, community-based home nursing institutions were responsible for providing the majority of visits (52.6%), followed by metropolitan (18.4%) and local (17.3%) hospitals, academic center-affiliated nursing institutions (9.8%) and community health centers (0.9%).

The total number of physician visits in 2004 was 14,118, with the most frequent by physicians contracted with home care nursing institutions (n = 7,024, 49.8%), followed by local community hospitals (21.3%), metropolitan hospitals (18.2%), academic medical centers (9.6%) and community health centers (1.1%). When looking at the nursing visits, home nursing care institutions accounted for the highest nursing visits (53.0%), followed by metropolitan hospitals (17.8%), local community hospitals (17.6%), academic medical centers (10.7%) and community health centers (0.9%). When categorized by resource utilization groups, the total number of nursing visits in 2004 was 28,253, with RUG-2 being the most frequent visits type among healthcare facilities (n = 16,247, 57.5%), while RUG-4 contributed the least (n = 1,395, 4.9%) (Table 2).

**Table 2:** Distribution of home healthcare professional visits under NHI by contracted category of healthcare facilities in 2004.

	No. of Physician visits (%)	No. of Nursing visits (%)				
		RUG*-1 ^†^	RUG-2 ^‡^	RUG-3^§^	RUG-4^||^	Total
Academi medical center	1,357 (9.6)	475 (1.7)	1,682 (6.0)	730 (2.6)	147 (0.5)	3,034 (10.7)
Metropolitan hospitals	2,570 (18.2)	470 (1.7)	2,986 (10.5)	1,317 (4.6)	248 (0.9)	5,021 (17.8)
Local community hospitals	3,006 (21.3)	588 (2.1)	2,740 (9.7)	1,409 (5.0)	243 (0.9)	4,980 (17.6)
Community health center	161 (1.1)	57 (0.2)	118 (0.4)	53 (0.2)	16 (0.06)	244 (0.9)
Home nursing care institutions	7,024 (49.8)	1,369 (4.8)	8,721 (30.9)	4,143 (14.7)	741 (2.6)	14,974 (53.0)
Total	14,118 (100)	2,959 (10.5)	16,247 (57.5)	7,652 (27.1)	1,395 (4.9)	28,253 (100)

*RUG: resource utilization group, please refer to text for details; ^**†**^RUG-1: patients who need common nursing care; ^‡^RUG-2: patients who need one additional kind of the special nursing care; ^§^RUG-3: patients who need two additional kinds of the special nursing care; ^||^RUG-4: patients who need three or more additional kinds of the special nursing care.

The skilled nursing service distributions in different healthcare facilities are summarized in Table 3. Of a total of 25,357 services, tube replacement, including the changing of nasogastric tubes, Foley catheters and tracheostomy tubes, accounted for 95.0% (n = 24,091). Wound care was the second most frequent service (n = 1,160, 4.6%) followed by ostomy care (n = 57, 0.2%). Intravenous fluid injection was the least frequently used service (n = 49, 0%) among all skilled nursing services.

**Table 3:** Distribution of total 25,357 home skilled nursing services under NHI in 2004 by contracted category of healthcare facilities.

Accreditation level	Tube replacement n (%)	Wound care n (%)	Ostomy care n (%)	IV fluid injection n (%)	Total n (%)
Academic medical center	1,718 (6.8)	133 (0.5)	2 (0)	0 (0)	1,853 (7.3)
Metropolitan hospitals	4,034 (15.9)	190 (0.7)	3 (0)	6 (0)	4,233 (16.7)
Local community hospitals	4,411 (17.4)	203(0.8)	37 (0.1)	31(0.1)	4,682 (18.5)
Community health center	189 (0.7)	15 (0.1)	1 (0)	0 (0)	205 (0.8)
Home nursing care institutions	13,739 (54.2)	619 (2.4)	14 (0.1)	12 (0)	14,384 (56.7)
Total	24,091 (95.0)	1,160 (4.6)	57 (0.2)	49 (0)	25,357 (100)

## Discussion

Population ageing and its impact on healthcare systems are important issues worldwide [1,2,5]. Aging and disabled populations need continual care delivered in the home or community healthcare institutions [3,14]. In Taiwan, home healthcare is reimbursed by the NHI system, a social health insurance that covers almost all of the country's citizens. The present nationwide survey revealed that 19,483 beneficiaries over 65 years of age received home healthcare services in 2004. However, the estimated number of older people who were disabled and in need of home personal or health care was 40,000-100,000 in 1993 [14], and is expected to grow to 118,000-240,000 by the end of 2010 [14,15], indicating that the patient number receiving home healthcare in 2004 under the NHI was much lower than the estimated disabled older population. There are several possible reasons which may account for this discrepancy. First, in Taiwan, home healthcare services are reimbursed by the NHI, which defines clear criteria of the application for home healthcare services [7]. However, the estimation of the disabled population was made according to the disability level evaluation by activities of daily living, or instrumental activities of daily living. Secondly, the study did not include the older population who needed living support such as personal care, home help, living care, or meal services. Lastly, the study did not include patients who received long-term care in long-term care facilities or in nursing homes.

Several studies have demonstrated the gender differences in healthcare utilization [16-19]. Women take more responsibility and have greater concern for their health conditions, visit their physician more often and utilize primary care services more than do males [18,19]. In this study, similar gender differences in the prevalence and utilization of home healthcare services were noted; females were more likely to use home healthcare services and receive more visits than males, even after adjustment for age. This may reflect the fact that there were more disabled females than males in these aged populations [15].

Community home nursing care institutions are one kind of nursing institution in Taiwan supplying home healthcare visits and services [20], and are regularly inspected by the national DOH. In this study, half of home healthcare services, as well as professional visits, were provided by these institutions. However, the quality of home healthcare provided by different healthcare institutes, and the outcome of patients who receive home healthcare from these different institutes, deserves further evaluation.

Physician-based comprehensive home visitation is effective in preventing functional decline, nursing home admission, and death in elderly people [21-23], and also is associated with good satisfaction among patients and their family members [24]. Taiwan NHI regulations limit physician home healthcare visits to one every two months [7]. To improve the outcomes of patients receiving home healthcare and the quality of home healthcare, a physician-based comprehensive and integrated home visit program seems to be necessary [25].

Taiwan's NHI pays for home nursing visits at a fixed rate based on resource utilization group classifications of patients, which is dependent on skilled nursing services. This payment system might contribute to the high rate of tube placement in home healthcare receivers and the potentially inappropriate intubation of these patients is possible. In this study, RUG-2 contributed to nearly 60% of all nursing visits. Previous studies revealed that the prevalence of indwelling urinary catheters or nasogastric tube insertion among long-term care facility residents in Taiwan is higher than that in the United States and European countries [26-29]. Long-term placement of urinary catheters or enteral feeding through nasogastric tubes can cause patient discomfort and lead to complications such as tube dislodgement, mucosal injury, infection and pressure ulcers, resulting in a decreased quality of life [26,30]. The rate of tube replacement of home healthcare patients in Taiwan deserves more attention.

The strengths of this study include its large sample size and the use of a complete nationwide computerized database representing the current practice pattern over a year. Although the study was a nationwide claims-based study, it also had some limitations. First, the one-year cross-sectional survey could not provide information about trends in home healthcare use. Second, the claims data did not provide detailed demographic and socio-economic data, or medical backgrounds of the beneficiaries, which precluded analysis of possible contributing factors such as education level, economic background, caregiver status, family composition, polypharmacy and inappropriate prescription for home healthcare utilization.

## Conclusions

The NHI program provides available, accessible and accountable home healthcare services for disabled older patients in Taiwan. However, only a limited number of disabled older patients apply for these services. Among these patients, individuals aged 75-84 years old account for the greatest proportion of total visits, and females used these services more than males, mainly for treatment in connection with cerebrovascular diseases. Most of the home healthcare services are provided by home nursing care institutions and the majority of home skilled nursing services involve tube replacement. Further research is needed to clarify the rate of tube replacements in home healthcare services in the elderly population in Taiwan.

## List of abbreviations

ICD-9-CM: International Classification of Disease, Ninth Revision, Clinical Modification; RUG: Resources Utilization Group; NHI: National Health Insurance; NHRI: National Health Research Institutes; WHO: World Health Organization.

## Competing interests

The authors declare that they have no competing interests.

## Authors' contributions

HTC conceived and carried out this study, performed the statistical analyses, interpreted the findings and drafted the manuscript. HYL participated in the design of this study and helped to interpret findings. IHH conducted the data mining and operated on the NHI dataset. HMM helped to draft the manuscript and interpreted the findings. SJH participated in the study design and coordination of the study, and helped to draft the manuscript. All authors have read and approved the final manuscript.

## Pre-publication history

The pre-publication history for this paper can be accessed here:

http://www.biomedcentral.com/1472-6963/10/274/prepub
